# Colitis-Associated Carcinoma: The Quintessential Epithelial Neoplasia Driven by Chronic Inflammation

**DOI:** 10.3390/cells15050481

**Published:** 2026-03-06

**Authors:** Michael G. Drage, Mari Mino-Kenudson

**Affiliations:** Mass General Brigham, Harvard Medical School, Boston, MA 02114, USA; mminokenudson@mgb.org

**Keywords:** inflammatory bowel disease (IBD), ulcerative colitis (UC), crohn’s disease (CrD), primary sclerosing cholangitis (PSC), colorectal dysplasia, carcinogenesis, chronic colitis, colitis-associated carcinoma (CAC)

## Abstract

Colitis-associated carcinoma (CAC) represents ~1% of colorectal carcinomas and has important differences from sporadic colorectal carcinoma (sCRC). The precursors and carcinomas that arise in the setting of IBD are uniquely challenging to visualize by endoscopy and diagnose via histology, and the rising prevalence of IBD amplifies the challenges of surveillance to informed management. Although in broad strokes, CAC and sCRC share molecular features (~85% chromosomal instability pathway 15% microsatellite instability high (MSI-H)), CAC has a distinct distribution of molecular abnormalities, including lower frequencies of APC and KRAS mutations, greater prevalence of IDH1R132H, and more frequent copy number alterations (e.g., MYC amplifications), and functional data indicate that most CACs show far less dependence on Wnt signaling than sCRC, suggesting a distinct pathogenesis from the earliest stages. Although there are significant gaps in our knowledge of the pathogenesis of CAC, our understanding is growing. This review summarizes how chronic colitis reshapes epithelial homeostasis and somatic evolution, resulting in the distinctive pathogenesis of CAC, and highlights knowledge gaps that could be addressed by applying multimodal technologies to well-annotated clinical material. The review is structured in two sections, the first introducing the IBDs and the homeostatic mechanisms that preserve integrity and prevent colorectal neoplasia. The second section compares failure modes in sporadic and colitic settings and describes the differences in the resulting neoplasms.

## 1. The Colon in Illness and Health

### 1.1. The Inflammatory Bowel Disease(s)

Inflammatory bowel disease (IBD) comprises a heterogenous spectrum of phenotypes that are unified by idiopathic, chronically dysregulated inflammation of the gastrointestinal tract. Patients may suffer disease limited to the tubal gut or additionally experience extraintestinal manifestations (EIMs) which can affect nearly any organ [1]. There are three clinical entities with distinct natural histories: ulcerative colitis (UC), Crohn’s disease (CrD), and IBD with comorbid primary sclerosing cholangitis (PSC), termed PSC-IBD. UC is the most common and is characterized by inflammatory activity predominantly limited to the colonic mucosa/superficial submucosa, tends to initiate in the rectum and progress proximally in a circumferential and continuous fashion, and is curable by total abdominal proctocolectomy. CrD can occur anywhere in the GI tract in segmental and/or discontinuous fashion and is characterized by inflammation that involves the mucosa and all layers of bowel wall, causing submucosal muscularization and hyperplasia of nerves, submucosal and myenteric fibrosis, and serosal fat wrapping. Manifestations of CrD vary amongst patients; some have predominantly fibrosing/stenosing disease, some have predominantly fistulizing disease, and others have a mixed pattern. A subset of CrD patients have perianal fistulizing disease, which is often difficult to manage [2]. Mucosal inflammation appears histologically similar between UC and CrD, with a subset of the latter showing non-necrotizing granulomas. PSC-IBD is the least common type (~8% of all IBD) and resembles UC with an atypical anatomic distribution, often arising in the ascending colon without involvement of the rectum. The IBDs are thought to arise due to a triggering inflammatory episode in predisposed individuals, but the determinants of predisposition are poorly understood. Alterations of the microbiome are associated with IBD, but it is unclear whether dysbiosis is a cause or consequence of the altered mucosal immune system. Interestingly, patients with IBD are enriched for carriers of colibactin-producing colonic flora [3], which has recently been found to be associated with the incidence of sCRC in young patients without IBD [4]. Although hundreds of IBD risk alleles have identified, only a small fraction of the risk (estimated to be less than 10%) is attributed to heritable genetic factors [5].

The mucosal sites involved by IBD are at increased risk of dysplasia and carcinoma, which is roughly equivalent for patients with UC and CrD with equal lengths of colon involved [6]. Although the risk appears to be decreasing thanks to improvements in therapy and advanced endoscopic techniques [7], the relative risk of neoplasia for patients with CrD and UC remains 1.9–2.6 and 2.4–2.7, respectively [8], with an overall 20% lifetime risk of CAC [9]. For patients with IBD, the independent risk factors for colorectal neoplasia include comorbid PSC, smoking, the duration of disease and length of colonic involvement, and most pertinent to the subject at hand, persistent neutrophilic inflammation. Surveillance endoscopy is a major component of patient care; all patients with UC proximal to the rectum and CrD involving at least 1/3 of the colon are recommended to undergo surveillance endoscopy every 1–3 years, beginning 8 years after diagnosis [10]. PSC-IBD has twice the risk of advanced colorectal neoplasia, and dysplasia is more often endoscopically invisible [11], warranting annual surveillance colonoscopy immediately upon diagnosis. The reasons for the enhanced risk of neoplasia in PSC-IBD are not yet clear.

### 1.2. The Human Colon

The human intestine is the second largest epithelial compartment in the body, (>30 m^2^ [12]), and by far the dirtiest. Many of the functions of the colon (and the small intestine) are performed by six fundamental types of mature epithelial cells: absorptive enterocytes (termed colonocytes in the colon) (responsible for nutrient uptake in the small intestine and primarily water reclamation in the colon); M cells (antigen sampling of the intestinal lumen); Paneth cells and goblet cells (produce antimicrobial peptides and mucin, respectively, both important for microbiome management as well as facilitating passage of stool); enteroendocrine cells (sensing luminal metabolic contents and influencing the metabolism and motility of the gastrointestinal tract); and lastly, the poorly understood tuft cells, which sense luminal metabolites and pathogens, and serve as signaling intermediaries between the epithelium and mucosal immune system [13]. These six morphologic types have been further refined via single-cell RNA sequencing (scRNAseq) into tentative functional subcategories beyond the scope of this review [14,15]. The intestinal mucosa must efficiently absorb select nutrients and water and assiduously exclude and simultaneously cooperate with the dense and complex commensal microbiome incubating within the lumen, whilst destroying and/or expelling the diverse array of pathogens that have evolved to infect or parasitize the gastrointestinal tract. These seemingly contradictory functions are accomplished by exquisitely choreographed dynamics between the intestinal epithelium, the commensal microbiome, the lamina propria immune system, the autonomic nervous system, the mesenchymal cells, vasculature, and extracellular matrix that comprise the tubal gut. The interactions that govern mucosal function in health and disease continue to be a subject of intense scrutiny.

### 1.3. Genomic Maintenance of the Colonic Epithelium

**Figure 1:**
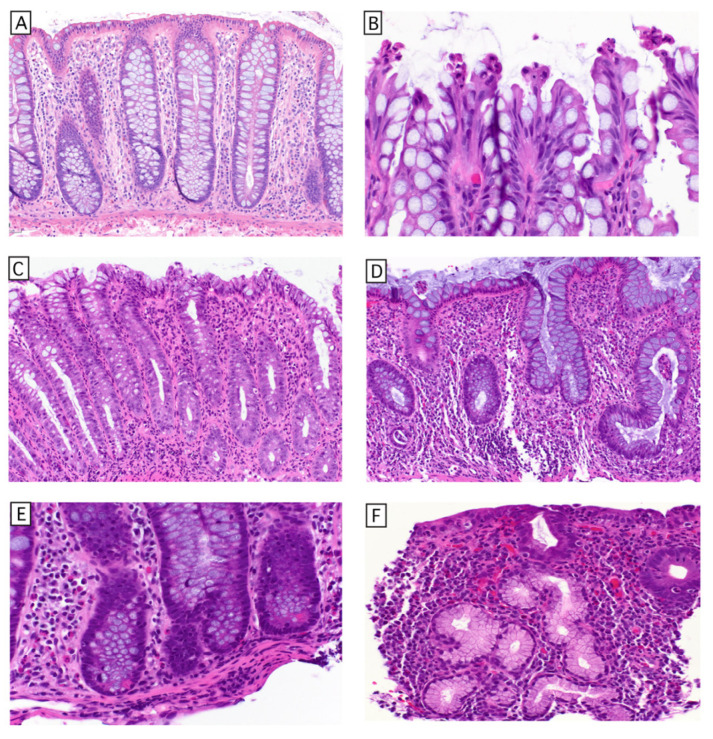
Histology of normal colonic mucosa and mucosal alterations of chronic injury. (**A**) Well-oriented section showing left colon crypt architecture with parallel crypts, each of which nearly touch the muscularis mucosae. In the left colon, epithelial differentiation is biased toward goblet cells, the lamina propria inflammatory cells are less dense than in the proximal colon, and Paneth cells are not present. The crypt base houses the CBC-scs, and the lymphatics deep to the crypts are not typically prominent on H&E stain (**B**) The epithelium at the surface undergoes apoptosis in the intercrypt space (**C**) Acute infectious colitis results in a luminally-oriented exudative inflammation that does not result in distorted crypt architecture (**D**–**F**). Features of chronic colitis include deep crypt branching, basal plasmacytosis and crypt atrophy/distortion, as well as epithelial metaplasia (trans-differentiation of pre-existing epithelium into a lineage not normally found at that location), attributed to epigenetic modifications driving adaptive and/or maladaptive gene expression changes. In the colon, the most frequent metaplasia is Paneth cell metaplasia (**E**). Pyloric metaplasia (**F**) is more frequently seen in the small intestine but also occurs in colonic mucosa.

Considering the seemingly conflicting demands placed on the colonic epithelium—its large surface area, high epithelial proliferation rate, and single epithelial layer separating the mucosal immune system from an enormous quantity and variety of proinflammatory stimuli, it is amazing that many of us never develop CRC. A few complementary structural and regulatory features mitigate this risk. First, the adult colon contains approximately 15 million crypts of Lieberkühn (henceforth called ‘crypts’) (Figure 1A), involutions of epithelium comprising approximately 2000 cells, creating a protected niche for the stem cell compartment at the base, physically removed from the digestive, immune, and microbial dynamics which occur at the luminal surface. Because the epithelial cells outside of this protected niche are indeed exposed to numerous injuries intrinsic to their environment, their rapid turnover (with an average epithelial lifespan of approximately 3–5 days) [16] serves a complementary mechanism to maintain genomic integrity by limiting the time for any single somatic cell to accumulate damage. Phylogenetic analysis of single nucleotide variants in crypts suggests that crypts undergo fission to expand the colon (annual fission rate of ~0.136) until approximately 20 years of age, at which point crypt fission slows to a steady state of ~0.00413 fissions per year for the rest of adult life [17].

### 1.4. Cell Competition Determines and Reinforces Colonic Epithelial Differentiation

Epithelial differentiation is determined by cell competition, a homeostatic process that maximizes the quality of somatic cells [18]. At the crypt base, LGR5+ stem cells divide symmetrically to produce identical daughter cells [19], which compete for limited space within this niche. This population of multipotent stem cells (henceforth referred to as crypt base columnar stem cells (CBC-scs)) is maintained by neighboring lymphatic endothelial cells and RSPO3+/GREM1+ fibroblasts [20]. These niche-supporting cells secrete WNT and R-spondin 3 (Rspo3), the latter of which potentiates Wnt signaling, as well as GREM1 and GREM2, which inhibit bone morphogenic protein (BMP) produced by other mesenchymal cells within the lamina propria. This environment restricts the stem cell compartment to the crypt base. Stem cells within the crypt base niche are supported by Paneth cells (in the right colon) or REG4+ deep secretory cells (in the left colon) [21], providing trophic and metabolic support via growth factors and lactate, respectively. Lactate serves as a substrate for oxidative phosphorylation [22], enabling the stem cells to produce both ATP and critical synthetic intermediates in the form of four- and five-carbon molecules that enter anabolic pathways to support cell growth/division (cataplerosis) [23]. Stem cells that are pushed out of the crypt base become short-lived transit-amplifying cells, which proliferate and then differentiate into mature epithelial cells as they move toward the surface, with differentiation enforced by an increasing gradient of BMP. The gradient of BMP is generated by the mesenchymal cells that surround the crypts, with basal trophocytes producing Grem1 (inhibiting BMP), and superficial telocytes producing BMP [24]. The first cell fate decision during epithelial differentiation is made between two basic lineages: 1. secretory (Paneth, goblet, enteroendocrine, and tuft cells) and 2. absorptive (colonocytes and M cells). This fate decision is also determined by cell competition in the form of lateral inhibition. Cells that commit to the secretory lineage express and secrete Notch ligands, which activate Notch receptors in adjacent cells. Notch activation induces transcriptional programs characteristic of absorptive cells and simultaneously suppresses Notch ligand expression in those neighbors. This lateral inhibition results in the characteristic alternating pattern of secretory and absorptive lineages in a roughly 1:1 ratio seen in the physiologic state (Figure 1A). Mature cells meet their end in the intercrypt space, where they undergo apoptosis and are mechanically extruded into the lumen by the advancing cells behind them [25] (Figure 1B). This process has long been thought to be triggered by cell crowding [26], but recent cell-tracking experiments in the murine small intestine provide compelling evidence for a competitive cell tension-based mechanism of extrusion [27]. While there are other mechanisms outside the scope of this review [28], it is clear that cell competition works in concert with morphogen gradients and mechanical forces [29] to create and maintain the diversity and spatial organization of the crypt epithelium, preserve the identity and function of each epithelial cell and ensure their appropriate removal whilst maintaining epithelial barrier function. Another benefit of cell competition is the passive elimination of aneuploid cells, a frequent occurrence in rapidly dividing stem cells [30].

### 1.5. Tissue Alterations of Chronic Colitis

Being a mucosal site frequently challenged by infectious organisms, the colon is designed to handle occasional acute episodes of active inflammation. In acute colitides, inflammation tends to be surface-/luminally directed, and more exudative, most importantly without basal plasmacytosis or alteration of the crypt architecture (Figure 1C). The chronic injury of IBD induces microscopic alterations in the intestinal mucosa that are an important component of the clinicopathologic diagnosis. Microscopic features of chronic colitis include crypt architectural distortion, deep crypt branching, basal plasmacytosis, and epithelial metaplasia (transdifferentiation of cells into a lineage not normally present at that location) (Figure 1D–F), features present in approximately 75% of patients who report symptoms of 4 months or longer duration [31,32,33]. The conventional teaching is that the architectural distortion reflects the relapsing/remitting course of IBD, due to active injury occurring to regenerating epithelium, and other processes causing chronic intermittent injury (diverticular disease, chronic ischemia, radiation therapy, medication effect such as immune checkpoint inhibition (which can sometimes be the trigger, unmasking IBD in predisposed patients [34], chronic graft-versus-host disease, anastomotic sites, and occasionally chronic bacterial colitides), all of which can result in the same histologic alterations (for an excellent review see [35]). IBD is a progressive disease, and over time, these changes become more pronounced, creating diagnostic challenges for pathologists (discussed later).

## 2. How the System Breaks Down: Neoplastic Pathways of sCRC and CAC

### 2.1. Two Common Pathways to sCRC

**Figure 2:**
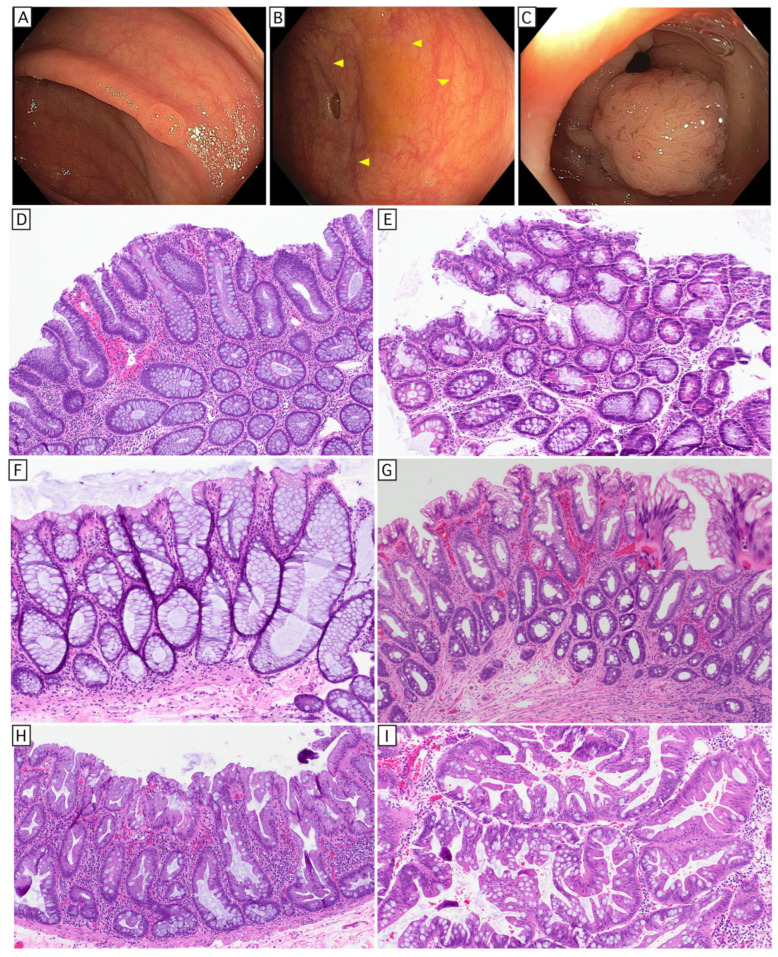
Macroscopic and microscopic features of common sporadic polyps. (**A**) Tubular adenomas and hyperplastic polyps are indistinguishable by endoscopy, (**B**) SSAPs are flat and covered by an altered mucus cap (arrowheads), and (**C**) TSAs are characterized by a pinecone-like appearance on endoscopy. Histologic appearance of tubular adenoma shows involved crypts comprise immature-appearing epithelium (**D**); sporadic adenomas occasionally show Paneth cell differentiation (**E**). Hyperplastic polyp, goblet cell-rich type (**F**) are characterized by swollen crypts with differentiation skewed toward goblet cells. Microvesicular hyperplastic polyps (**G**) show greater serration of the upper crypt and immature proliferative compartment at the crypt base and microvesicular cytoplasm (inset). SSAPs (**H**) also show microvesicular cytoplasm but instead show expansile growth at the crypt base. TSAs (**I**) show a complex villous growth pattern with elaborate serration and minimal nuclear atypia.

The vast majority (~85%) of colorectal carcinomas follow the chromosomal instability (CIN) pathway. These are initiated by truncating mutations of APC [36] that result in B-catenin stabilization and constitutively increased WNT signaling [37], enabling persistent stemness and escape from the stem cell niche [38]. The success of APC-mutant adenomas to overtake their crypt (an achievement referred to as ‘crypt fixation’) depends on their secretion of NOTUM, a WNT inhibitor, which shuts down the competitive proliferation of adjacent wild-type CBC-scs and induces their terminal differentiation [39]. Continuous Wnt signaling produces a localized increase in crypt fission [40], resulting in polypoid expansion visible on endoscopy (Figure 2A), a precursor referred to as an adenoma (Figure 2D). Alterations of the MAPK pathway such as KRAS/BRAF are often early mutational events in sporadic adenomas. Subsequent alterations in TP53 and SMAD4, allelic losses of tumor suppressors via CIN, and a growing predominance of oncofetal gene expression signatures are associated with progression to carcinoma [41]. Surprisingly, studies in murine models suggest that even though they also harbor oncogenic alterations in KRAS and TP53, invasive carcinomas remain dependent on Wnt signaling, as restoration of APC leads to growth arrest and terminal differentiation, even in lesions growing in extracolonic locations [42]. This persistent dependence on Wnt signaling is not seen in other carcinomas, such as breast and non-small cell lung carcinoma, where alterations in TP53 and KRAS mitigate WNT dependence [43].

In the United States, about 10–15% of colorectal carcinomas arise via the serrated pathway, which originate from a prototypical BRAF-mutant precursor called a sessile serrated adenoma/polyp (SSAP) (Figure 2H). Not driven by Wnt signaling, SSAPs lack the immature morphology of adenomas, and tend to grow as a flat lesion that is endoscopically appreciable due to an altered mucus cap (Figure 2B). The SSAP is thought to arise from a differentiated cell that undergoes gastric metaplasia, and is tightly associated with a missense mutation in valine 600 of the BRAF gene, leading to constitutive activation [44]. SSAPs tend to occur in the right colon and progress via an epigenomic alteration called CpG island methylator phenotype (CIMP), which drives evolution via methylation of CpG islands in the promoters of tumor suppressor genes. For instance, CpG island methylation of the MLH1 promoter leads to loss of MLH1 (and consequently PMS2) expression, resulting in functional inactivation of the mismatch repair (MMR) complex. Loss of MMR function results in accumulation of insertion/deletion mutations (indels), driving a rapid molecular evolution. The accumulating indels generate a tumor mutational burden (TMB) orders of magnitude higher than that seen in CRC arising via the CIN pathway, producing abundant tumor-specific neoantigens and frequently inciting a pronounced cytotoxic inflammatory response. Presumably due to this inflammatory response, MSI-H carcinomas tend to have a more indolent natural history and are enriched for cancers that respond to immune checkpoint inhibition therapy. Less commonly, perhaps ~1% of sCRC arise from a traditional serrated adenoma (TSA). In contrast to the flat SSAP, these are exophytic/polypoid growths with a fractal villous and serrated architecture, granting it a pinecone-like appearance on endoscopy (Figure 2C), also initiated by deregulated MAPK signaling. Approximately 50% are associated with BRAF mutation (some of which arise from SSAP); an additional 30% are associated with KRAS mutations, which tend to occur in the left colon, demonstrate more abundant goblet cell differentiation, and can give rise to invasive carcinomas that maintain a serrated pattern [45,46,47]. Approximately 3% of CRCs are due to inherited cancer predisposition syndromes, which will not be discussed.

A single-cell sequencing study [48] has confirmed that the morphologic differences between adenomas and SSAPs correlate with a lack of Wnt signaling in the SSAPs, whose gene expression profile is compatible with their morphologic features: a hybrid of goblet cells and gastric foveolar epithelium (Figure 2H). They also found that neoplasms with either BRAF or KRAS mutations had a greater cytotoxic inflammatory response compared to normal colon and APC-deficient tumors that arose from APC loss in stem cells. This study included an experiment in a murine model that compared the immune response to neoplasms initiated by loss of APC in a stem cell compartment vs. the more difficult to induce tumors caused by APC loss in a non-stem cell. Although the resultant tumors showed similar morphology, the neoplasms that initiated from loss of APC in the non-stem cell compartment were associated with a greater cytotoxic immune response (CD8, NK cells, and gamma-delta T cells), suggesting an additional feature of stemness beyond Wnt signaling, an important determinant of the immune response to the tumor. Tumors from these lesions developed scattered regional stem cell-like expression profiles, and these regions were relatively exclusive of cytotoxic infiltrate, which other work suggests may be due to methylation of Wnt antagonists [47]. This correlation suggests that relative differences in stem cell-like expression profiles are an important determinant of the tumor immune response independent of neoantigen burden. A similar correlation has been observed in hepatocellular carcinoma (HCC), where beta catenin-mutant HCC has a greater tendency to exclude CD8 T cells and evade anti-PD-1 therapy [49]. Although the precise mechanism is unknown, a compelling hypothesis is that the immune system has evolved mechanisms to spare stem cell compartments from its most destructive effectors.

CACs express low levels of the stem cell marker LGR5 [50] and exhibit a transcriptional profile distinctly absent of Wnt signaling [51], so one might expect these tumors to be susceptible to cytotoxic inflammation. The limited data available suggest otherwise. A comparison of the tumor immune microenvironment of CAC (*n* = 24) and sCRC (*n* = 48) (both groups including ~15% MSI-H cases) found that CAC had less infiltration of CD3, CD8, FoxP3 and PD-L1-positive cells [52], and another study found that CD8 T cells did not express granzyme B (a measure of dysfunction) [53], suggesting that CAC may suppress cytotoxic immunity via mechanisms other than ‘stemness’.

### 2.2. Pathogenesis of IBD-Related Epithelial Neoplasia

In IBD patients, active neutrophilic inflammation detected by histology of mucosal biopsies is the single most important predictor of all major adverse outcomes, including dysplasia and carcinogenesis [54,55], and the complete absence of mucosal neutrophils by histology is undergoing prospective evaluation as a primary treatment target (VERDICT; NCT04259138) [56]. Neutrophil-derived reactive oxygen species (ROS) induce oxidative DNA damage, mitochondrial depolarization, lipid peroxidation, and the unfolded protein response in epithelial cells, evidenced by measurable increases in aldehydes such as trans-4-hydroxy-2-nonenal (HNE), which forms DNA adducts at levels at 3- to 28-fold higher than those in uninflamed mucosa [57]; murine and human colitic tissue have increased 8-oxoG (a direct measure of oxidative DNA damage), more prevalent double-strand breaks, and the amount of histologic inflammation correlated with the prevalence of 8-oxoG and gamma-H2AX-positive epithelial cells [58]. While there is abundant evidence that neutrophils induce epithelial injury, it is becoming increasingly clear that IL17-producing CD8+ T cells are also important mediators of epithelial injury [59]. Within the epithelium, the NFκB family of transcription factors integrate signaling from TNFα, IL-1, and toll-like receptors via interactions with their downstream signaling pathways. Initially, NFκB initially propagates pro-inflammatory signaling pathways while simultaneously driving an adaptive antioxidant response via NRF2/KEAP1, but persistent NF-kB signaling in the epithelium increases turnover of ZO-1 and occludin, compromising epithelial barrier function [60], sensitizes epithelial cells to cell death [61] and leads to altered Wnt signaling, resulting in excessive stem cell proliferation and impaired differentiation of secretory lineages (goblet cells and Paneth cells), further compromising epithelial barrier function [62,63]. Persistent NFκB signaling can also trigger aberrant activation of activation-induced cytidine deaminase (AID), whose function is normally reserved for the hypermutation of immunoglobulin genes or those of infectious viruses (along with APOBEC), here playing a role in accelerating mutagenesis more generally [64]. APOBEC enzymes are rarely active in normal epithelium; one recent deep sequencing study of thousands of crypts found only two crypts (one ileal and one colonic) with mutational signatures indicative of APOBEC3a activity in an older patient without IBD. The same study found an average of 3000 substitutions and 300 indels, including a single cancer driver mutation (e.g., PIK3CA, ERBB2, ERBB3, and FBXW7), in approximately 1% of normal crypts from 50-to 60-year-old individuals without IBD. Importantly, each cancer driver mutation clone was limited to 1–2 crypts, showing no evidence of selection (in which case one would expect the clone to spread across more crypts) [65].

The epithelial injury and crypt destruction of IBD results in a dramatic increase in crypt turnover. While healthy adults replace ~0.4% (50,000) of crypts per year, segments involved by IBD undergo 2.16 fissions per crypt per year [17], accelerating somatic evolution of the epithelium. Whole-exome sequencing studies performed on isolated crypts from densely sampled rectal mucosa from patients with ulcerative colitis and age-matched controls demonstrated that IBD patients had a high prevalence of expanded clones harboring non-synonymous mutations in ARID1A, FBXW7, NFKBIZ, PIGR, ZC3H12A, and genes in the IL17 and TLR pathways, consistent with positive selection. Interestingly, the signaling pathways of the mutated genes converge on either NFKBIZ or related signaling molecules (PIGR and ZC3H12A) in a mutually exclusive manner, all sharing a predicted effect: loss of IL17 signaling in the colonic epithelium [66]. These mutations were highly prevalent in IBD patients (53–83% of the rectal mucosa replaced by clones with one or more mutations in this pathway, one clone covering 19 cm^2^) and were absent in non-IBD controls. Interestingly, mutations in this pathway are distinctly absent from CAC, indicating negative selection for carcinogenesis [17,65].

Approximately 15–20% of CACs arise as multifocal synchronous lesions [67,68], as opposed to 3–5% of sCRCs [69], raising the question of whether multifocal lesions are clonally related (arising from large clonal fields with cancer driver mutations) or distinct carcinogenic events. Previous studies have found pathogenic mutations in TP53 in nondysplastic mucosa fields [70,71], giving rise to the concept that nondysplastic colonic mucosa can carry mutations in TP53. Since our morphologic conceptualization of dysplasia has evolved since those publications, it is possible that some of these ‘non-dysplastic’ clones may be previously unrecognized forms of dysplasia. For instance, Figure 1B of [72] may represent an area now recognized as goblet cell-deficient dysplasia. Recent work by Chatila et al. [51] used targeted exon sequencing and whole-exome sequencing to compare pathogenic driver mutations in background mucosa to dysplastic lesions and carcinomas from patients with multiple synchronous lesions, making two important observations: 1. There were no oncogenic mutations in non-dysplastic mucosa. 2. Multifocal dysplasia and carcinomas from the same patient had predominantly unique pathogenic mutations in p53, and carcinomas had unique structural abnormalities. Synchronous but unique alterations in p53 indicate convergent evolution. Convergent evolution in somatic tissues has been observed in other settings [73], and appears to be a reproducible feature of chronically injured mammalian tissue, suggesting a strong context-dependent selection for pathway-specific alterations. The tendency to develop multiple synchronous independent neoplastic transformation events indicates that the entire segment of mucosa involved by chronic inflammation reaches a state of critical vulnerability to neoplastic transformation at approximately the same time, but that the determinants of the risk of neoplasia are not conventional cancer driver mutations and may instead be epigenetic.

While inflammation can explain the elevated mutation rates, the principle of cell competition suggests an explanation why CAC seems to follow a reverse molecular pathogenesis. Work in murine systems has demonstrated that CBC-sc competition is neutral under physiologic conditions, and that KRAS G12D mutation resulted in a selective advantage (25% above neutral), resulting in the mutant cells displacing wild-type cells over time. In contrast, TP53 mutant stem cells demonstrated neutral competition with wild-type stem cells under physiologic conditions but showed a selective advantage (8% above neutral) in the setting of DSS colitis [74]. Thus, while KRAS might offer a proliferative and/or survival advantage under physiologic conditions, p53 mutations offer a survival advantage in the setting of oxidative injury from chronic inflammation, which may explain the prevalence of p53 mutations in early neoplastic lesions, and the tendency for these mutations to accumulate specifically in the involved segment of the colon in patients with ulcerative colitis [75]. It remains unknown why PSC-IBD has a pronounced risk of neoplasia. A recent multiparameter analysis of a relatively small cohort of patients using single-cell transcriptomics and flow cytometry identified a pathogenic epithelial antigen-specific CD4+/FoxP3+/IL17A+ T cell response that appears specific to patients with PSC [76], a possible culprit of their increased rate of colorectal neoplastic progression. Altered bile acids are also a potential contributing factor, and in support of this hypothesis, liver transplantation has been shown to reduce the risk of subsequent colorectal neoplasia in patients with PSC-IBD [77].

### 2.3. Macroscopic and Histologic Features of IBD-Neoplasia

IBD-related colorectal neoplasia is enriched for uncommon features that make it challenging to detect and diagnose. For visible lesions, a gastroenterologist is tasked with describing the endoscopic abnormality according to standardized criteria [78], to document the completeness of resection and risk of recurrence. Approximately 1/3 of dysplastic lesions are flat, subtle lesions that are poorly visualized by endoscopy, even with advanced techniques, and approximately 20% are endoscopically invisible, even in highly specialized tertiary referral centers using state-of-the-art techniques [79].

The challenge of macroscopic and microscopic evaluation of CAC was first noted in the seminal work by Dawson and Pryse-Davies [80], who reviewed colectomies from 663 patients with UC performed over an approximately 12-year timespan; the prevalence of carcinoma was 2.9 percent. In the carcinoma cases, there was often no visible macroscopic lesion, or only a subtle mucosal irregularity or stricture was noted. Histologically, the mucosa adjacent to the carcinomas showed a spectrum of histologic alterations, with some regions showing unequivocal dysplasia, others appearing purely inflammatory, and many ‘intermediate forms’ that were challenging to classify.

Although the intervening decades have improved our understanding of the molecular pathogenesis of IBD-associated neoplasia, these insights have not yet led to major advances in diagnostic practice. This is reflected in the most recent consensus classification manuscript, which echoes Dawson and Pryse-Davies: “In this study, we utilized the term ‘dysplasia’ for all lesions despite the fact that some do not necessarily exhibit traditional cytologic features of neoplastic precursor lesions” [81]. As described, IBD-related dysplasia has a variety of forms that can co-exist within one lesion and can be difficult to differentiate from background changes in non-dysplastic epithelium. The histologic features of these lesions are also subtle. Despite their subtle alterations and often minimal nuclear atypia, amongst the flat/invisible dysplasia, 41% of low-grade and 93% of high-grade dysplasia are aneuploid [82], whereas only 8% of polypoid low-grade IBD-related dysplasia and 9% of sporadic adenomas are aneuploid [83]. Numerous studies have described morphologies and proposed criteria to subclassify nonconventional dysplasia in IBD, with changing terminology making comparisons somewhat difficult [84,85,86].

**Figure 3:**
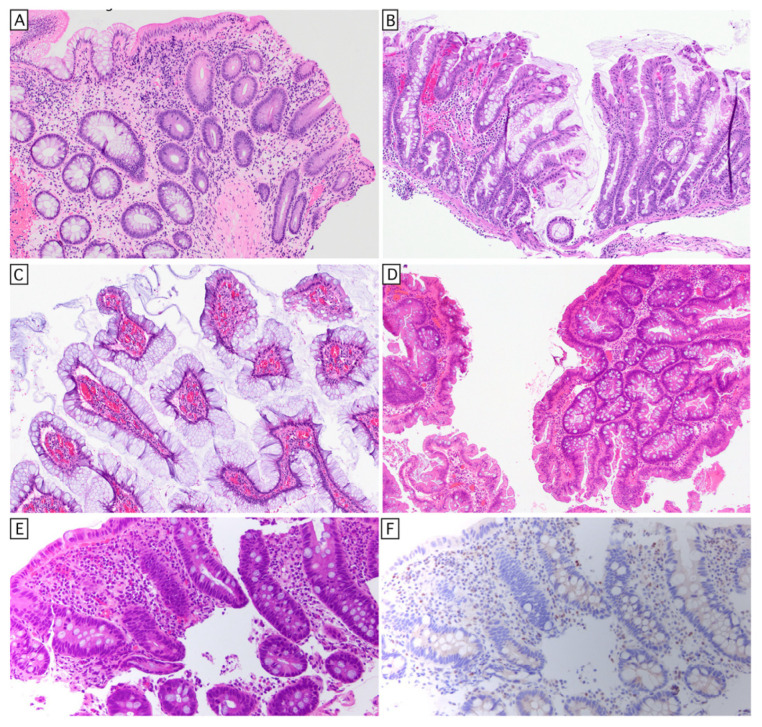
Histology of IBD-associated dysplasia and carcinoma. (**A**) Goblet cell-deficient dysplasia (right half of image). (**B**) Serrated dysplasia, NOS. (**C**) Gastric (aka hypermucinous) dysplasia, low grade, superficial aspect. (**D**) TSA-like dysplasia, which resembles conventional TSA but tends to have more pronounced lateral growth instead of the discrete pedunculated polyps seen in the conventional setting. (**E**) Crypt cell dysplasia characterized by nuclear hyperchromasia and slight enlargement at the base of the crypts; (**F**) p53 immunostain shows mutant (null) expression pattern in the same field of view with preserved expression in lamina propria cells.

Some commonly agreed non-conventional histologic classifications include crypt cell dysplasia, goblet cell-deficient dysplasia, hypermucinous (aka gastric) dysplasia [87], TSA-like dysplasia, and serrated dysplasia, NOS (Figure 3). Some authors separately distinguish sessile serrated-like dysplasia from serrated dysplasia, and dysplasia with increased Paneth cell differentiation from conventional dysplasia. In our experience, increased Paneth cell differentiation seems to be not uncommon in sporadic adenomas (Figure 2E). Of non-conventional dysplasia, hypermucinous/gastric, crypt cell, and goblet cell-deficient dysplasia appear to be high-risk subtypes for progression, but whether this is attributed to differences in biology or superior interobserver concordance is unknown. These subtypes are often endoscopically invisible, more frequently aneuploid and/or KRAS mutant, and show a higher risk of progression [86]. Interestingly, these three histologic subtypes represent greater than half of the dysplastic lesions in patients with PSC-IBD, with crypt cell dysplasia being the most common subtype in these patients [88].

Because individual lesions often show mixed features, most studies show high interobserver disagreements in classification amongst experienced subspecialty-trained experts [89]. While awareness of the histological subtypes facilitates their recognition and diagnosis, the histological subtype is of no clinical import. Determinants of the endoscopic surveillance interval and/or colectomy are the endoscopic assessment of completeness of resection and the histologic grade of dysplasia [90]. One important caveat is that for some of the villous dysplasia subtypes, the histologic grade is best determined by the deep aspects, which are not always sampled in mucosal biopsies. Since the biology remains incompletely understood, the current classification of histologic subtypes is best considered a work in progress.

**Figure 4:**
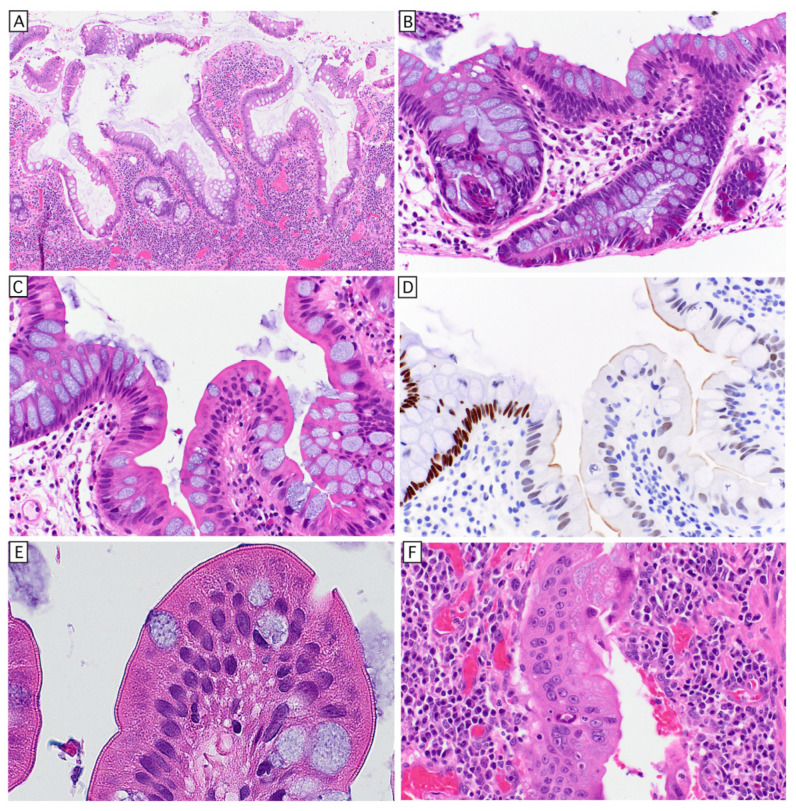
IBD-associated epithelial alterations in long-standing UC. Characteristic alterations of long-standing colitis include (**A**) villiform transformation (**B**) hyperplasia of enteroendocrine cells in non-neoplastic mucosa, (**C**–**E**) small intestinal metaplasia with villous growth loss of SATB2 expression (**D**), and a well-developed brush border (**E**). (**F**) Multinucleation in regenerative epithelium.

Longstanding IBD remodels the mucosal architecture (sometimes referred to as villiform transformation; Figure 4A) and results in alterations of epithelial morphology suggestive of aberrant/indeterminant differentiation with hybrid features. These alterations may range from hyperplasia of certain cell types (Figure 4B) to clear abnormalities in cell morphologies, with varying degrees of differentiation along other lineages (Figure 4C–F). The histology suggests epigenomic instability, which is likely propagated not only by cell-intrinsic epigenomic abnormalities, but also abnormalities of cell–cell and cell/matrix signaling and as discussed, aberrant dynamics of cell competition. The relationship of these histological changes to the somatic evolution towards lack of IL17 signaling with/without anti-apoptosis mutations, such as mutations in TP53, to the development of dysplasia/carcinoma is not well understood. A more thorough understanding of the non-neoplastic alterations in IBD may complement our understanding and classification of these challenging neoplastic precursors.

## 3. Conclusions

The preponderance of evidence suggests that the chronic active inflammation in IBD selects for epithelium that does not respond to IL17 signaling and occasionally selecting anti-apoptotic alterations such as loss of p53 function (at a much lower rate). The lack of IL17 pathway mutations in CAC suggests that these are independent mechanisms granting a selective advantage to the colonic epithelium context of IBD, and that cells blind to IL17 signaling tend not progress to carcinoma, while the p53 mutant clones survive but also engender copy number variants that are important for progression to carcinogenesis. Whether these alterations initiate in stem cells or differentiated cells is unknown, but regardless, the morphology and transcriptional profile of dysplastic lesions in IBD suggests an aberrant version of mature epithelium with hybrid features, distinctly lacking in ‘stemness’ both morphologically and as defined by absent Wnt signaling. While this review focuses on the colon, the general principle that chronic inflammation and/or injury causes aberrant cell competition likely applies to other tissue sites, each with tissue- and injury-specific nuances. Our growing understanding of the mechanisms of competition and its failure modes has the potential to lead to interventions that can reduce the incidence of neoplasia and potentially increase healthy lifespan.

For these recent discoveries to impact clinical care of patients with IBD, our understanding of the mechanisms needs to be more refined than our current vague sense that loss of identity puts tissues at risk for neoplasia. Identifying key rate-limiting steps in the progression to neoplasia, invasive behavior, and metastasis remains an important goal, as does improving surveillance modalities to identify tissue that requires removal [91]. More generally, determining the most appropriately achievable treatment target should reduce the risk of neoplasia at the population scale [56]. In addition to presenting an important management challenge, the heightened risk of neoplasia in patients with PSC-IBD presents an opportunity for comparative studies to better understand risk factors of colonic neoplasia, some of which may be distinct from active neutrophilic inflammation. It is our belief that the complex questions presented here will be best approached by the multimodal, spatially informed technologies that are becoming increasingly available [92]. We have attempted to highlight some recent advances from single-cell sequencing studies, but spatially informed multiomics will be able to provide more granular information about the relationships between cells as they collaborate to define the behavior of the system (whether healthy or unhealthy). Well-designed studies using human tissue that has been thoroughly annotated for clinicopathologic features will accelerate translation from basic science to improvements in healthcare.

## Data Availability

No new data were created or analyzed in this study.
